# *In-silico* and structure-based assessment to evaluate pathogenicity of missense mutations associated with non-small cell lung cancer identified in the Eph-ephrin class of proteins

**DOI:** 10.5808/gi.22069

**Published:** 2023-09-27

**Authors:** Shubhashish Chakraborty, Reshita Baruah, Neha Mishra, Ashok K Varma

**Affiliations:** 1 Advanced Centre for Treatment, Research and Education in Cancer, Kharghar, Navi Mumbai, Maharashtra 410210, India; 2 Homi Bhabha National Institute, Training School Complex, Anushaktinagar, Mumbai, Maharashtra 400094, India

**Keywords:** erythropoietin producing hepatocellular (Eph), Eph receptor-interacting protein (Ephrin), mutations, structural alterations

## Abstract

Ephs belong to the largest family of receptor tyrosine kinase and are highly conserved both sequentially and structurally. The structural organization of Eph is similar to other receptor tyrosine kinases; constituting the extracellular ligand binding domain, a fibronectin domain followed by intracellular juxtamembrane kinase, and SAM domain. Eph binds to respective ephrin ligand, through the ligand binding domain and forms a tetrameric complex to activate the kinase domain. Eph-ephrin regulates many downstream pathways that lead to physiological events such as cell migration, proliferation, and growth. Therefore, considering the importance of Eph-ephrin class of protein in tumorigenesis, 7,620 clinically reported missense mutations belonging to the class of variables of unknown significance were retrieved from cBioPortal and evaluated for pathogenicity. Thirty-two mutations predicted to be pathogenic using SIFT, Polyphen-2, PROVEAN, SNPs&GO, PMut, iSTABLE, and PremPS *in-silico* tools were found located either in critical functional regions or encompassing interactions at the binding interface of Eph-ephrin. However, seven were reported in non-small cell lung cancer (NSCLC). Considering the relevance of receptor tyrosine kinases and Eph in NSCLC, these seven mutations were assessed for change in the folding pattern using molecular dynamic simulation. Structural alterations, stability, flexibility, compactness, and solvent-exposed area was observed in EphA3 Trp790Cys, EphA7 Leu749Phe, EphB1 Gly685Cys, EphB4 Val748Ala, and Ephrin A2 Trp112Cys. Hence, it can be concluded that the evaluated mutations have potential to alter the folding pattern and thus can be further validated by *in-vitro*, structural and *in-vivo* studies for clinical management.

## Introduction

Receptor tyrosine kinase (RTKs), a sub-class of tyrosine kinase, regulates numerous physiological events such as cell growth, division, metabolism, and motility. Genomic alterations are one of the major reasons for RTK signaling dysregulation associated with cancer. Mutations reported in different functional domains of RTKs are linked to the kinase constitutive expression, ligand-independent signaling, and drug sensitivity [1].

Eph receptor belongs to the largest family of RTK. Based on sequence homology, Eph and ephrin have been classified into EphA/EphrinA and EphB/EphrinB class of families [2]. So far, nine EphA, five EphB, five EphrinA, and three EphrinB have been reported. Eph and ephrin are membrane-bound and thus regulate cell-cell interaction, migration, partitioning, and cell adhesion [3]. Eph-ephrin as a family is ubiquitously expressed in different tissues [4]. Structurally Eph receptors are similar to RTKs, comprises extracellular region, a ligand binding and fibronectin repeat domain, whereas intracellular has a juxtamembrane, kinase, and SAM domain. The extracellular domain facilitates binding to ephrin. The juxtamembrane domain has two highly conserved tyrosine that regulate activation of catalytic core of the receptor. Furthermore, the kinase and SAM domains allow the binding of other cellular proteins. The overall domain organization of EphAs and EphBs are similar, however, ephrin A is GPI anchored to the membrane, whereas ephrin B has an intracellular PDZ binding domain. The Eph-ephrin complex follows an exclusive signaling pattern wherein either Eph activates Ephrin for reverse signaling, or Ephrin activates Eph for forward signaling [4]. A trans-interaction of Eph-ephrin followed by heterotetramer formation activates the signaling cascade and transforms the kinase domain from closed to open conformation which in-turn, expose the ATP binding pocket [5]. Eph-ephrin regulates many downstream signaling pathways, such as phosphoinositide 3-kinase and Ras mitogen-activated protein kinase that control cell morphology, migration, division, differentiation, and proliferation. Dysregulation of these physiological events can initiate or drive tumor formation, and therefore Eph-ephrin has been reported to act both as a tumor promoter and suppressor [6]. Differential expression of Eph-ephrin has been reported in different cancers; however, only a few documented information regarding the effect of mutations on the varying expression pattern of Eph-ephrin have been reported. Recently, we have reported the structure of three EphA7 mutants, Gly656Arg, Gly656Glu, and Asp751His [7]. To our observation, these missense mutations affected the intramolecular interactions, transforming the secondary structures and critical functional regions that can further interfere with ATP binding and catalytic activity of kinase [7].

Mutational landscape studies of Eph-ephrin have documented 7,620 mutations as per the information available in cBioPortal cancer database [8,9]. Eph is sequentially and structurally conserved to epidermal growth factor receptor (EGFR) and insulin-like growth factor receptor (IGFR) [10]. Genetic alterations in EGFR, IGFR, and anaplastic lymphoma kinase have already been linked to oncogenic transformation in non-small cell lung cancer (NSCLC) [11]. A crosstalk between EGFR and EphA2 has also been related to resistance against known tyrosine kinase inhibitors [12]. EphA2 overexpressed cells displayed increased expression of EGFR mRNA whereas lowered expression of ephrin-A1 mRNA. EGFR-EphA2 increased expression is correlated with poor prognosis and response to cetuximab in stage IV and Ras wt colorectal cancer patients [13]. siRNA mediated knockdown of EphA2 or treatment with ephrinA1-Fc revert the erlotinib and gefitinib (reversible tyrosine kinase inhibitors) resistance in NSCLC [14]. Increased EphA2 mRNA and protein in afatinib resistant NSCLC cells were also correlated to high S897 phosphorylation [15,16]. However, most Eph mutations remain uncharacterized and referred to as variables of unknown significance (VUS). Structural and functional validation of this vast pool of mutations is a challenging task for scienists. Therefore, an attempt has been made through *in silico* based approach to evaluate mutations with a higher probability of deleterious effect at protein level. In recent reports, we have predicted the pathognectiy of the mutations identified in secretory clusterin [17], BRCA2 [18], and RSK1 [19]. All the missense mutations reported in the Eph family independent of the cancer type were filtered using *in silico* prediction tools. These tools rely on algorithms based on criteria such as residue conservation, substitution, position, and stability. Mutations explored based on pathogenicity score obtained using *in silico* tools were further scrutinized with respect to their location in the functional domain, and also intra/inter molecular interactions. We obtained 32 mutations that were predicted to be pathogenic as well as located in key functional regions and classified them as 'mutations of interest'. Among these 32 mutations, seven were reported in NSCLC. Hence, noting the mutational predomainance and relevance, we further evaluated these seven mutations by structural and molecular dynamics (MD) simulations.

## Methods

### Retrieval of mutations to predict pathogenicity

Mutations were retrieved from the cBioPortal, a large-scale cancer genomic public database. Missense mutations being predominant, were selected for *in silico* based pathogenicity analysis. Functional assessment of missense mutations were evaluated in the form of pathogenicity scores using mutation assessor, sorting intolerant from tolerant (SIFT), and Polyphen-2. The mutation assessor scores, residual change considering the sequential conservation with sequence homologs, and furthermore clustering the sequences into subfamilies. Finally, it scores the mutation based on global and subfamily level conservation [20]. The functional impact score is classified into four classes. A score of 0 or less is labeled as neutral, score 0—1.99 as low, score 2—3.49 as a medium, and a score of 3.50 and above as high. A mutation with a high score can effect protein function. SIFT is another server that indicates the functional effect of a mutation, considering the sequence conservation of substituted residue and physical properties of the amino acid. Apart from assigning the impact score, it also predicts the confidence of the result to prevent any inaccuracy. A SIFT score of zero indicates the deleterious impact of mutations; a score above zero refers to tolerated and scores for which the server is not definite referred to as low confidence. Polyphen-2 uses two approaches: multiple sequence alignment-based conservation analysis and structure-based prediction. Structure-based prediction is based on parameters such as accessible surface area, hydrophobic propensity, and B-factor. A score below 0.5 denotes mutation being benign, whereas a score above 0.95 denotes probably damaging [21].

Furthermore, three additional pathogenicity prediction tools were used to check the pathogenicity of mutants. PROVEAN (Protein Variation Effect Analyzer) analyzes sequence-based conservation using semi-global pairwise sequence alignment [22]. For binary analysis, a default score cut of –2.5 was used, wherein mutations scoring below –2.5 were classified as deleterious and above –2.5 neutral. PMut relies on sequence conservation and changes in the physiochemical nature imparted by mutations. The PMut result True, indicates that the mutation is pathogenic, and False indicates benign/tolerated. [23]. SNPs&GO, which combines results from PANTHER and PhD-SNP. SNPs&GO predicts the pathogenicity of a mutation based on sequence conservation, function, and structure of the mutant [24]. The output algorithm classifies a mutation as disease-associated or neutral. Additionally, ConSurf was used to identify highly conserved residues with location, i.e., whether it is buried or exposed in the protein's three-dimensional conformation [25]. The scores obtained from the mutation assessor, SIFT, Polyphen-2, PROVEAN, PMut, and SNP&GO were compared, and the mutants scoring deleterious in all or a minimum of three were selected for stability analysis.

### Stability prediction of probable pathogenic mutants

Mutations with a higher probability of being pathogenic were assessed for change in the stability at the protein structure level. Protein Data Bank (PDB) sequence from PDB ID: 3HIL and 2QBX, corresponding to the SAM domain of EphA1 and ligand binding domain (LBD) of EphB2, respectively, has a different sequence number than the reported original FASTA sequence which may be due to presence of additional residues during crystallization. Therefore, sequence number correction was done to prevent any errors. PDB ID: 3HIL had a difference of ten residues and PDB ID: 2QBX had a difference of eight residues. Furthermore, iSTABLE and PremPS were used to analyze ΔΔG. iSTABLE is a meta predictor that combines the result from I-Mutant and Mupro, where sequence-based analysis is used to predict ΔΔG. PremPS calculates ΔΔG using protein structure which helps to create an accurate dataset. For both the servers ΔΔG > 0 indicate increased stability, whereas ΔΔG < 0 indicates decreased stability. Predicted pathogenic mutations showing ΔΔG score of ≥0.8 and ≥2 in iSTABLE and PremPS, respectively were set as threshold after comparing the overall ΔΔG score across the Eph-ephrin family. EphA6 has not been included in the study as sequence-related ambiguity was found for most of its mutations.

### Folding pattern analysis of Eph-ephrin

Intramolecular and intermolecular interactions were analyzed using Ligplot+ and PDBsum. Mutations located in critical functional regions such as nucleotide-binding region, hinge region, catalytic loop, and activation loop were selected for pathogenic prediction. Residues present in the binding interface of Eph-ephrin, ATP-Kinase domain, SAM-SAM domain, and Eph dimer/tetramer were selected for MD analysis. Residues located other than the functional regions or binding interface but interacting with critical residues essential for folding analysis were also selected.

### Molecular dynamic simulations of Eph-ephrin wild-type and mutants associated with NSCLC

GROMAS 2018.1 with OPLS-AA/L force field was used to carry out molecular dynamic simulation [26,27]. TIP3P water model and counter ions were used for system solvation and neutralization. Initial energy minimization was performed using the steepest descent algorithm with a 1,000 kJ/mol/nm tolerance. Further, the system was equilibrated using NVT (constant number of particles, volume, and temperature) and NPT (constant number of particles, pressure, and temperature) for 100 ps each. The temperature was set 300K using Berendsen thermostat [28] with a pressure of one bar.

PDB files of Eph and ephrin structures were obtained from the RCSB protein data bank. Mutations in structure files were incorporated using chimera software [29]. Further, the equilibrated system was subjected to 100 ns of the production run for Ephrin A2, 250 ns for EphB1 and 200 ns for all other Eph’s with time step integration of 2 fs. The trajectories were saved every two ps, and root mean square deviation (RMSD), root mean square fluctuation (RMSF), radius of gyration (Rg), solvent accessible surface area (SASA), and secondary structure were analyzed using Gromacs 2020.4.

## Results and Discussion

### Most of the pathogenic mutations identified on Ephs located at the kinase and ligand binding domain

**Fig. 1.**
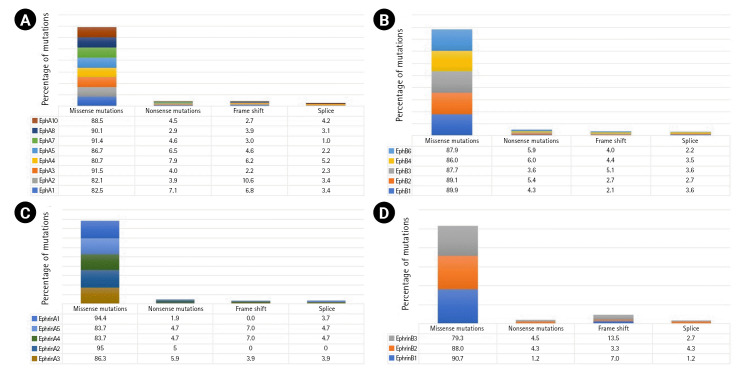
Mutational predominance in Eph A (A), Eph B (B), ephrin A (C), and ephrin B (D).

A total of 7,620 mutations across the Eph-ephrin family were retrieved from the cBioPortal database. Among these, 4,886 mutations were found in EphA, 2,182 in EphB, and 552 in the Ephrin subfamily. The maximum number of mutations reported were missense (Fig. 1) mainly VUS. The functional impact of mutations were analyzed using *in silico* based approach. Among the vast list of missense mutations, we found 25%–35% of mutations predicted to be pathogenic in the Eph family. However, the number in ephrin was much more diverse, with 28.9% of mutations predicted to be pathogenic in Ephrin A2 whereas 10.5% in Ephrin B1 (Supplementary Fig. 1). Missense mutations primarily affect the stability or folding pattern of a protein. Thus, analyzing the change in Gibbs free energy (ΔG) of mutations becomes essential. The difference in the folding pattern due to free energy change (ΔΔG) between wild-type and mutant structure is associated with the change in protein stability. ΔΔG value of the mutations was analyzed and compared using iSTABLE and PremPS. A total of 80 mutations were seen to have a ΔΔG value more than the threshold.

Mutations prevailing in the kinase and LBD have been associated with constitutive activation of the kinase domain. EGFR Leu858Arg mutation located in the kinase domain was reported to hyper-activate the kinase, leading to oncogenesis [30]. Similarly, fibroblast growth factor receptor (FGFR) Ser249Cys mutation dimerizes the receptor and leads to constitutive signaling by ligand-independent signal transduction [31]. To our observation, most of the predicted pathogenic mutations were located in the kinase and LBDs (Supplementary Fig. 2).

### Mutations identified at functional regions of Ephs and Ephrins influence receptor-ligand activity

We analyzed 80 residues showing a higher probability of being pathogenic and unstable among that 17.4% of these are located in N-lobe, whereas 82.6% are in the C-lobe of the kinase domain (Supplementary Table 1). N-lobe is primarily responsible for nucleotide-binding, and C-lobe regulates the catalytic activity [32]. The N-terminal helix and beta-sheet present in N-lobe possess weak intramolecular interaction to maintain the geometry of the nucleotide-binding groove. The nucleotide-binding groove facilitates phosphate entry near the nucleotide-binding loop, and thus any change in intramolecular interactions due to the mutation can affect ATP binding. EphA7 Arg676, located in the N-terminal helix, and EphB1 Gly685, situated on the beta-strand, can play a significant role in the formation of nucleotide-binding groove. Similarly, EphA3 Iso682, EphA5 Iso736, and EphA5 Iso737 are located in the loop connecting the N-terminal helix with the beta-sheet. The conformation of residues present in this loop and their intramolecular interactions modulate the groove geometry. Mutations identified in C-lobe were primarily located in the C-terminal helices. EphA2 Phe758 located in the highly conserved DFG motif, determines the conformation of kinases. EphB1 Val741, EphB1 His742, EphA3 Ala748, EphA3 Ala749, EphA5 His798, and EphA5 Ala802 are part of the catalytic loop. Catalytic loop recognizes substrate and assist its binding to conserved HRD motif [33]. EphB1 Val760 and EphA3 Val762 are present on the N-terminal of the activation loop whereas EphA3 Trp790 is a part of the activation loop. Activation loop forms a cleft for substrate binding and begins with a conserved DFG residue [33]. Mutations identified at or near the catalytic and activation loop can drastically compensate the catalytic efficiency of the kinase. The LBD has the second most identified number of mutations. LBD is rich in beta sheets, and mutations found across all the different beta strands, are not accumulated in a single subdomain, as observed in kinase domain. EphA5 Phe132, Phe132, and Phe132, EphA5 Tyr99, and EphA7 Iso68 are positioned and oriented near the interactive groove, and thus can influence Eph-ephrin interactions.

Further, we analyzed the intramolecular and intermolecular interactions prevailing within these residues. EphA2 Iso619 in the nucleotide-binding loop interacts with ATP, and modulates nucleotide-binding affinity within the kinase domain. EphA3 Val688 forms a hydrophobic interaction with Gly687 and Ala671 present near the nucleotide-binding groove and maintains the groove geometry. In the C-lobe, EphA5 Leu791, EphA7 Leu749, EphB4 Leu731, and EphB3 Leu749 forms hydrophobic interaction and hydrogen bonds with conserved tyrosine residue present in the catalytic loop. Thus, these intermolecular interactions maintain the molecular conformation of the conserved tyrosine residues and regulate phosphorylation. It has been reported that mutations interfering with the phosphorylation of conserved tyrosine residues promote tumor formation in prostate cancer [34]. Therefore, though these residues do not reside in functional regions, they can significantly influence catalytic activity. It has also been found that EphB4 Val748 forms a hydrogen bond with Gly699, Asn698, and hydrophobic interaction with Met696 present in the hinge region. The hinge region is a flexible loop connecting N-lobe and C-lobe to allow transition between the active and inactive conformation of kinases. Thus, a change in interaction patterns within the region affects transitions between the conformations. The LBD of Eph is responsible for interaction with ephrin and thus, residues involved in the binding interface are of great importance. EphA3 Phe152 forms non-bonded interactions with ephrin A5. Whereas, EphA4 Leu33 and Leu43 form non-bonded interactions with Phe136 and Arg135, respectively allowing formation of a homodimer essential for receptor activation. Additionally, EphA3 Iso109 was identified to be a crucial residue as it forms hydrophobic interaction with Arg104 and Leu111, which further interacts with the ephrinA5. However, in the ephrins class of protein, EphrinA2 Trp112 was found to interact through weak hydrophobic interaction with Ser58 of EphA4. Thus, a mutation in this residue can prevent interaction between EphA4 and ephrin A2.

From the 80 residues harbouring the predicted pathogenic and unstable mutations, a final 32 were found to be located at critical functional regions and possess essential interactions with residues that can influence receptor-ligand activity (Supplementary Table 2).

### Significant structural alterations have been observed in Ephrin A2 Trp112Cys and EphA7 Leu749Phe mutants

**Fig. 2.**
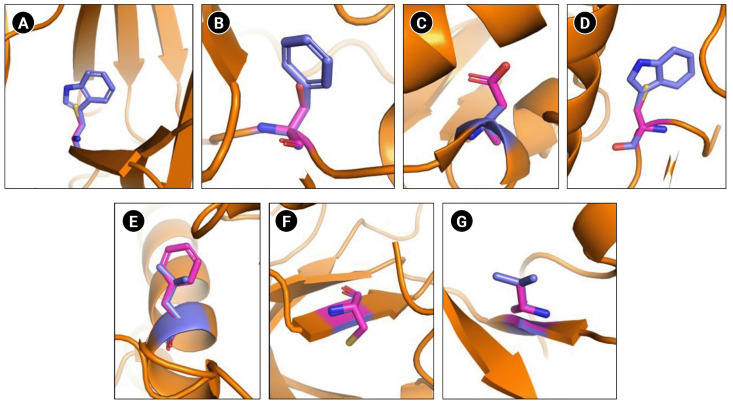
Schematic representation of wild type and mutated residues. (A) Ephrin A2 receptor binding domain Trp112Cys. (B) EphA3 ligand binding domain Phe152Ser. (C) EphA3 kinase domain Ala749Asp. (D) EphA3 kinase domain Trp790Cys. (E) EphA7 kinase domain Leu749Phe. (F) EphB1 kinase domain Gly685Cys. (F) EphB4 kinase domain Val748Ala. Wild type residues are indicated in blue and mutant in pink.

Among the 32 mutations found across different cancer types, seven mutations are reported in NSCLC (Fig. 2). Different reports suggest the crucial role of Eph in NSCLC wherein the expression pattern and activity varies with Ephs [12,35-42]. EphA7 was reported to be overexpressed and correlated to tumor proliferation in NSCLC [41]. In another study, silencing of EphA7 in A549 cells was reported to reduce cell viability, invasion, and metastasis [40]. Furthermore, mutations in RTKs have been linked to therapy resistance in NSCLC. EGFR Thr790Met mutation has been reported to be responsible for 60% of resistance against tyrosine kinase inhibitors. Similarly, multiple mutations in ALK—Leu1196Met, Leu1152Arg, Cys1156Tyr, Ser1206Tyr, and Gly1269Ala were reported to induce resistance to ALK inhibitors [11]. Thus, considering the importance of Eph in NSCLC, we analyzed these seven mutations for structural alterations using molecular dynamic simulation. To compare structural alterations perturbations due to the mutations RMSD, RMSF, Rg, SASA, and secondary structural changes were analyzed (Table 1).

### Effect of Trp112Cys mutation on Ephrin A2 (33-173 amino acids) structure

**Fig. 3.**
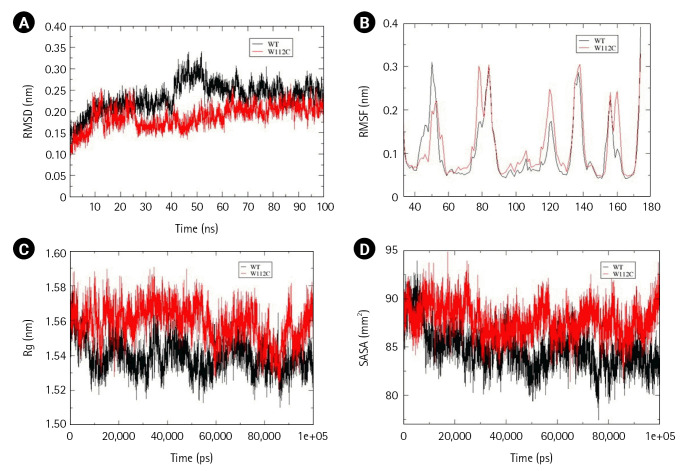
Assessment of change in structural stability and dynamics due to Ephrin A2 W112C mutation. (A) Background root mean square deviation (RMSD). (B) Root mean square fluctuation (RMSF). (C) Radius of gyration (Rg). (D) Solvent accessible surface area (SASA). Black indicates wild type whereas red indicates W112C mutation.

Within the Ephrin family, only one mutation Ephrin A2 Trp112Cys was reported in NSCLC and predicted to be pathogenic. Interestingly, the mutant structure attained stability at 30ns, whereas the wild-type at 60 ns of simulation. Time-averaged RMSD values for wild-type and mutant were calculated to be 0.234 nm and 0.188 nm, respectively, which suggest stabilizing effect of mutation over the Ephrin A2 structure. The time-averaged RMSF values of wild-type and mutant were 0.104 nm and 0.117 nm, respectively. It has been found that an amino acid stretch from 40–60 residues tend to attain lesser flexibility than the wild-type, whereas residues from 60–80, 90–130, and a small region across 160 residues gained higher flexibility than the wild-type. These calculations suggest that Trp112Cys mutation have local and global effect with respect to flexibility and an overall increase in the dynamic nature of the ephrin A2 receptor binding domain. No significant difference in Rg values was observed. However, the calculated SASA values for wild-type and mutant were 84.79 nm and 87.79 nm suggesting a slight disturbance in the hydrophobic core, allowing increased solvent-exposed surface area without compromising compactness of mutant structure (Fig. 3A–3D). Furthermore, to understand the overall change in secondary structure, define secondary structure of protein (DSSP) analysis was performed and found an increase in the coiled region in the mutant structure (Supplementary Table 3).

### Effect of mutations prevailing in kinase and LBD of EphA

**Fig. 4.**
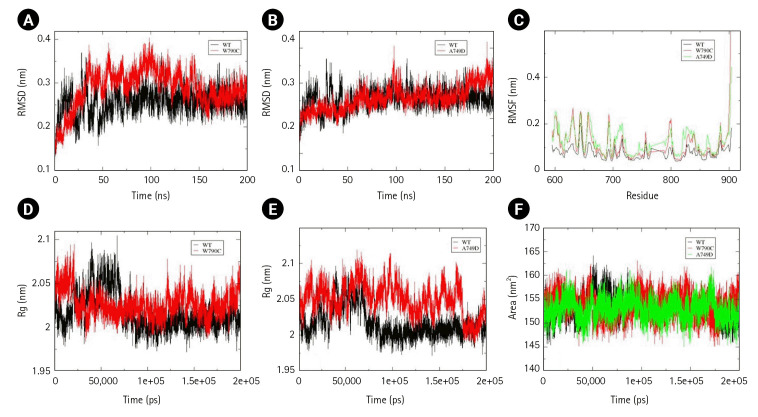
Assessment of change in structural stability and dynamics due to EphA3 W790C and A749D mutations. (A) Background root mean square deviation (RMSD) of W790Cys. (B) RMSD of A749D. (C) Root mean square fluctuation (RMSF) of W790C and A749D. (D) Radius of gyration (Rg) of W790C. (E) Rg of A749D. (F) Solvent accessible surface area of W790C and A749D. Black indicates wild type, red indicates W112C mutation, and green A749D mutation.

Two mutations, Ala749Asp and Trp790Cys, present in the kinase domain (577–947) and a mutation Leu749Phe present in the LBD (29–201) of EphA3 were assessed for structural changes. Unlike Ephrin A2, all the EphA were simulated till 200 ns as they attained stability post 100 ns of simulations. Slight decrease in conformational stability was calculated by the time-averaged RMSD values for Trp790Cys mutation. Time-averaged Rg value for Trp790Cys mutant and wild-type was calculated to be 1.6 nm and 2.03 nm, respectively, suggesting an increased structural compactness due to the mutation. No significant change in conformational stability, compactness, and solvent-accessibility area was observed for Ala749Asp mutation. However, local and global changes in the flexibility of the EphA3 kinase domain were observed due to both Trp790Cys and Ala749Asp mutations. No change was observed in SASA value for wild-type and Trp790Cys mutation (Fig. 4A–4F).

**Fig. 5.**
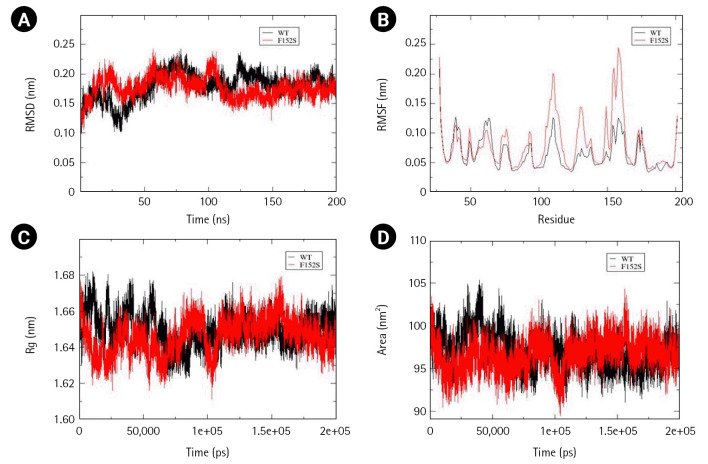
Assessment of change in structural stability and dynamics due to EphA3 F152S mutation. (A) Background root mean square deviation (RMSD). (B) Root mean square fluctuation (RMSF). (C) Radius of gyration (Rg). (D) Solvent accessible surface area. Black indicates wild type whereas red indicates F152S mutation.

EphA3 Phe152Ser mutation located in the LBD showed no substantial difference in the structure with respect to the calculated time-averaged RMSD, Rg, and SASA values (Fig. 5A, 5C, and 5D). However, a slight change observed in RMSF suggested a gain of flexibility in mutant structure over wild-type (Fig. 5B).

**Fig. 6.**
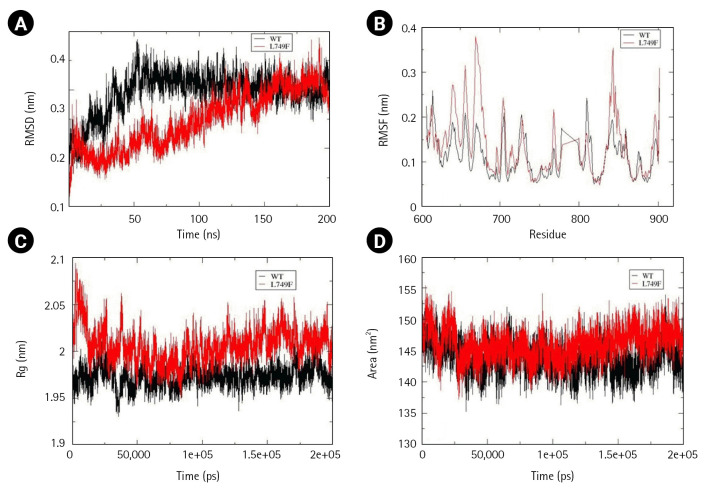
Assessment of change in structural stability and dynamics due to EphA7 L749F mutation. (A) Background root mean square deviation (RMSD). (B) Root mean square fluctuation (RMSF). (C) Radius of gyration (Rg). (D) Solvent accessible surface area. Black indicates wild type whereas red indicates F152S mutation.

Besides EphA3, a mutation Leu749Phe present in the kinase domain (590–899) of EphA7 was also analyzed. Time-averaged RMSD for EphA7 wild-type and Leu749Phe mutant were calculated to be 0.295 nm and 0.248 nm, which suggest mutant to be more stable than the wild-type. Regarding structural dynamics, time-averaged RMSF values calculated for EphA7 wild-type and Leu749Phe were 0.112 and 0.136, respectively, suggesting an increased dynamics of mutant structure. An increase in SASA vakue with time-averaged value of 143.7 nm^2^ for wild-type and 146.0 nm^2^ for mutant was observed. However, no change observed in Rg (Fig. 6A–6D) suggested an increased solvent accessibile area with no change in compactness. An increase in alpha helix in the mutant structure was also observed (Supplementary Table 3).

### Effect of mutations prevailing in the kinase domain of EphB

**Fig. 7.**
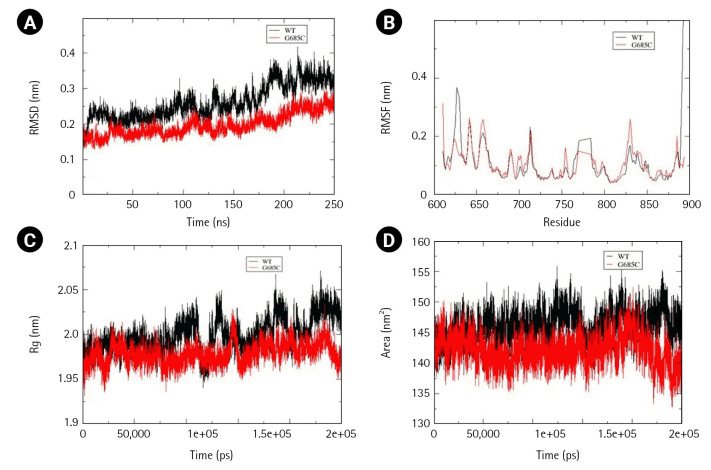
Assessment of change in structural stability and dynamics due to EphB1 G685C mutation. (A) Background root mean square deviation (RMSD). (B) Root mean square fluctuation (RMSF). (C) Radius of gyration (Rg). (D) Solvent accessible surface area. Black indicates wild type whereas red indicates G685C mutation.

Two mutations, Gly685Cys and Val748Ala, present in the kinase domain of EphB1 (602–896) and EphB4 (598–892) respectively, were analyzed for alterations in folding psttern. Unlike other Ephs, the wild-type structure of EphB1 was not stabilized at 200 ns and therefore the simulation was extended to 250 ns. In comparison to wild-type, mutant Gly685Cys showed less fluctuation with major difference observed between 180-190ns. However, time-averaged values of RMSD, RMSF, and Rg suggested no significant difference between the wild-type and mutant structure. Significant decrease in SASA value, was observed in mutant structure (Fig. 7A–7D).

**Fig. 8.**
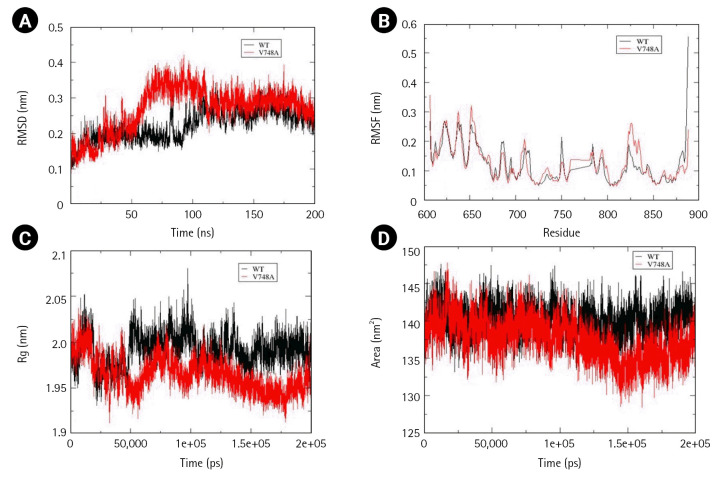
Assessment of change in structural stability and dynamics due to EphB4 V748A mutation. (A) Background root mean square deviation (RMSD). (B) Root mean square fluctuation (RMSF). (C) Radius of gyration (Rg). (D) Solvent accessible surface area. Black indicates wild type whereas red indicates V748A mutation.

Time-averaged RMSD for EphB4 and Val748Ala was calculated to be 0.224 nm and 0.269 nm, respectively, indicating that mutant structure less stable than the wild-type. Fluctuation in RMSD was observed between 50 to 100 ns, wherein mutant RMSD increases up to 0.4 nm, whereas wild-type RMSD decreases to 0.15 nm. Additionally, there was a decrease in solvent accessible area as determined by time-averaged SASA value wherein mutant SASA value calculated was 140.1 and that for wild-type was 137.5. No significant changes were observed in the calculated time average RMSF and Rg values (Fig. 8A–8D).

From cBioPortal 7,620 missense mutations in the Eph-ephrin family were evaluated for pathogenicity and stability using *in silico*, structural and MD-based approach. To our finding, maximum predicted mutations were located in the Eph receptor's ligand binding and the kinase domain. However, no such domain or region enriched with mutational pattern was observed in the ephrin ligand. Further, residues corresponding to the 80-point mutations were analyzed for their location and intra/intermolecular interactions. Thirty-two ‘mutations of interest’ were seen to alter critical functional and interacting regions of the receptor or ligand. However, seven—EphA3 Phe152Ser, Ala749Asp, Trp790Cys, EphA7 Leu749Phe, EphB1 Gly685Cys, EphB4 Val748Ala, and EphrinA2 Trp112Cys were reported in NSCLC. Considering the critical role of Eph in NSCLC, these were analyzed for structural changes through MD simulation. Change in the folding pattern of mutant protein was analyzed by calculating time-averaged RMSD, RMSF, Rg, SASA, and DSSP. Among the seven analyzed mutants, EphA3 Ala749Asp and EphA3 Phe152Ser located in the kinase and LBD, showed no drastic alterations at the structural level, suggesting a lesser probability of these mutations to perturb the 3D conformation of the protein.EphA3 Trp790Cys, located in the kinase domain, led to decreased conformational stability and increased compactness as suggested by the calculated time-averaged RMSD and Rg. Major alterations were observed for EphA7 Leu749Phe mutant situated in the kinase domain, wherein decreased SASA and increased conformational stability as well as flexibility was observed. Similar results were obtained for Ephrin A2 Trp112Cys mutant wherein increased stability but contrasting increased flexibility and solvent accessible area was observed. Two EphB mutations—EphB1 Gly685Cys and EphB4 Val748Ala located in the kinase domain showed decreased solvent-accessible surface with no significant change in structural compactness. Decreased conformational stability was observed for EphB4 Val748Ala mutant. In conclusion, this preliminary study categorized mutations reported in the Eph-ephrin family that can be potentially pathogenic specifically in the context of NSCLC and can be further validated through structural and functional studies.

## Figures and Tables

**Table 1.** Time-averaged structural parameters for wild type and mutants

	RMSD	RMSF	Rg	SASA
Ephrin A2	0.234	0.104	1.54	84.8
Trp112Cys	0.188	0.117	1.56	87.8
EphA3 (KD)	0.261	0.11	2.03	153.9
Ala749Asp	0.257	0.13	2.05	152.9
Trp790Cys	0.288	0.12	1.6	154.0
EphA3 (LBD)	0.17	0.065	1.65	96.3
Phe152Ser	0.17	0.08	1.65	96.6
EphA7 (KD)	0.295	0.112	1.97	143.7
Leu749Phe	0.248	0.136	2.01	146.0
EphB1 (KD)	0.26	0.104	2	146.5
G685C	0.25	0.104	1.98	141.8
EphB4 (KD)	0.224	0.123	1.99	140.1
V748A	0.269	0.125	1.96	137.5

RMSD, root mean square deviation; RMSF, root mean square fluctuation; Rg, radius of gyration; SASA, solvent accessible surface area.

## Supplementary Materials

Supplementary Fig. 1. Percentage of pathogenic mutations in ephrin A (A) and ephrin B (B).

Supplementary Fig. 2. Percentage of pathogenic mutations in functional domains of EphA (A) and EphB (B).

Supplementary Table 1. List of 80 mutations predicted to be pathogenic and altering protein stability.

Supplementary Table 2. List of 32 mutations for different cancer types.

Supplementary Table 3. Time-averaged secondary structure percentage analysis.
