# Supplementary figures and images for: In-silico and structure-based assessment to evaluate pathogenicity of missense mutations associated with non-small cell lung cancer identified in the Eph-ephrin class of proteins
Source: Genomics Inform. 2023 Sep 27;21(3):e30. doi: 10.5808/gi.22069 (PMC10584653; doi:10.5808/gi.22069)

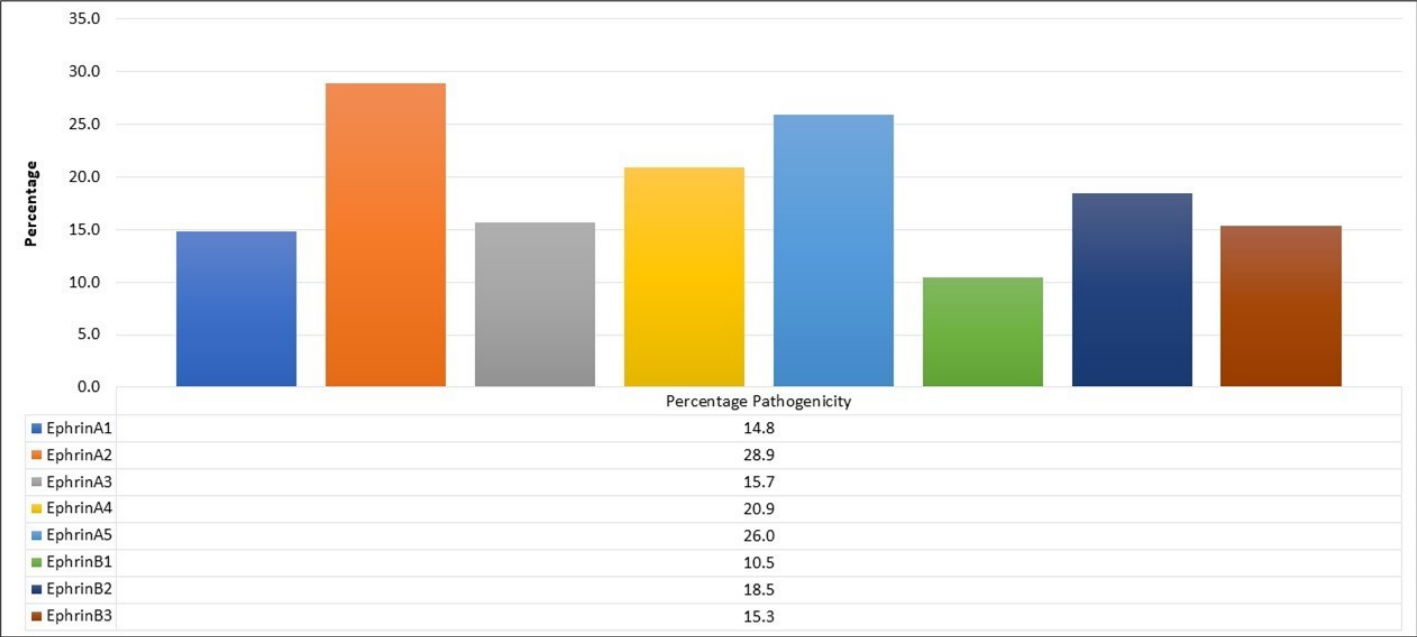

Supplementary figure 1 – Percentage of pathogenic mutations in (a) Ephrin A and (b) Ephrin B

Supplement: Supplementary Fig. 1. — Percentage of pathogenic mutations in ephrin A (A) and ephrin B (B). [file gi-22069-Supplementary-Fig-1.pdf]
