# Supplementary material for: In-silico and structure-based assessment to evaluate pathogenicity of missense mutations associated with non-small cell lung cancer identified in the Eph-ephrin class of proteins
Source: Genomics Inform. 2023 Sep 27;21(3):e30. doi: 10.5808/gi.22069 (PMC10584653; doi:10.5808/gi.22069)
Supplement: Supplementary Fig. 2. — Percentage of pathogenic mutations in functional domains of EphA (A) and EphB (B). [file gi-22069-Supplementary-Fig-2.pdf]

(a)

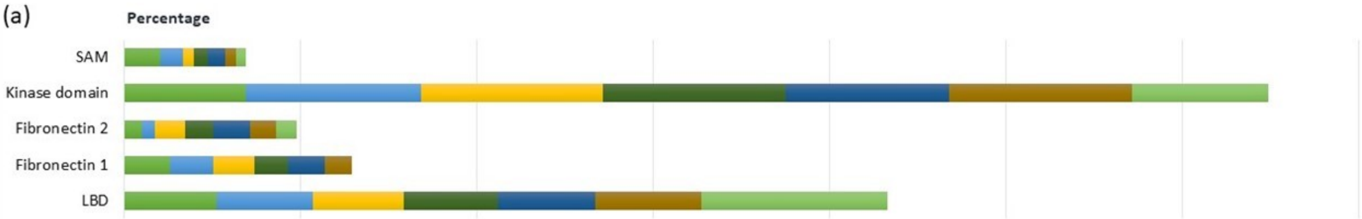

	LBD	Fibronectin 1	Fibronectin 2	Kinase domain	SAM
EphA1	26.2	13.1	5	34.3	10.1
EphA2	27.4	12.3	3.8	50.0	6.6
EphA3	25.7	11.7	8.5	51.3	2.9
EphA4	26.8	9.4	7.9	52.0	3.9
EphA5	27.5	10.5	10.5	46.4	5.1
EphA7	30.2	7.5	7.5	51.8	3.0
EphA10	52.9	0	5.7	38.6	2.9

(b)

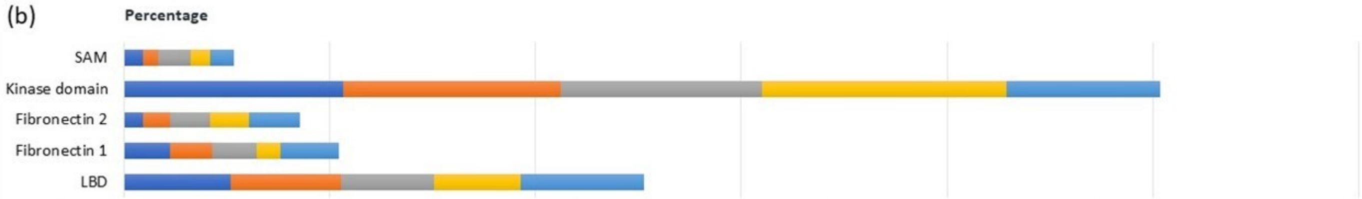

	LBD	Fibronectin 1	Fibronectin 2	Kinase domain	SAM
EphB1	25.9	11.3	4.7	53.3	4.7
EphB2	26.9	10.2	6.5	52.8	3.7
EphB3	22.5	10.8	9.8	49.0	7.8
EphB4	20.9	5.8	9.3	59.3	4.7
EphB6	30.0	14.2	12.5	37.5	5.8

Supplementary figure 2 - Percentage of pathogenic mutations in functional domains of **(a)** EphA and **(b)** EphB
