# Supplementary material for: In-silico and structure-based assessment to evaluate pathogenicity of missense mutations associated with non-small cell lung cancer identified in the Eph-ephrin class of proteins
Source: Genomics Inform. 2023 Sep 27;21(3):e30. doi: 10.5808/gi.22069 (PMC10584653; doi:10.5808/gi.22069)
Supplement: Supplementary Table 1. — List of 80 mutations predicted to be pathogenic and altering protein stability. [file gi-22069-Supplementary-Table-1.pdf]

**Supplementary Table 1 – List of 80 mutations predicted to be pathogenic and altering protein stability**					
**Protein**	**Residue**	**Position**	**Mutation**	**Domain**	**Subdomain**
EphA1	Val	918	Ala	SAM	Helix 1
EphA1	Tyr	930	Cys	SAM	Loop
EphA1	Phe	939	Tyr	SAM	Helix 3
EphA2	Trp	52	Gly	LBD	Loop
EphA2	Phe	410	Iso	Fibronectin 1	Beta 7 Fibronectin
EphA2	Leu	510	Pro	Fibronectin 2	Beta 6 fibronectin
EphA2	Iso	619	Ser	Kinase	beta 1
EphA2	Phe	758	Cys	Kinase	Loop
EphA2	Leu	836	Phe	Kinase	Loop
EphA2	Arg	861	Pro	Kinase	C-terminal Helix
EphA2	Phe	864	Leu	Kinase	C-terminal Helix
EphA3	Leu	33	Arg	LBD	Beta 1
EphA3	Val	73	Asp	LBD	Loop
EphA3	Iso	109	Asn	LBD	Helix 1
EphA3	Iso	146	Thr	LBD	Beta 9
EphA3	Phe	152	Ser	LBD	Helix3
EphA3	Iso	682	Asn	Kinase	Loop
EphA3	Val	688	Asp	Kinase	N-terminal Beta 4
EphA3	Leu	711	Arg	Kinase	C-terminal Helix
EphA3	Ala	748	Thr	Kinase	C-terminal Helix
EphA3	Ala	749	Asp	Kinase	C-terminal Helix
EphA3	Val	762	Gly	Kinase	C-terminal beta
EphA3	Trp	790	Cys	Kinase	Beta Hairpin
EphA3	Tyr	825	His	Kinase	Beta Hairpin
EphA3	Leu	843	Arg	Kinase	Loop
EphA3	Leu	856	Pro	Kinase	C-terminal Beta
EphA3	Trp	861	Ser	Kinase	C-terminal Helix
EphA4	Leuu	33	Ser	LBD	Beta 1
EphA4	Leu	43	His	LBD	Loop
EphA4	Cys	204	Ser	LBD	Beta 13
EphA4	Gly	251	Asp	Ectodomain	Beta Hairpin
EphA4	Cys	366	Gly	Fibronectin 1	Beta 3 fibronectin
EphA5	Tyr	99	His	LBD	Beta 3
EphA5	Phe	132	Val	LBD	Beta 7
EphA5	Leu	113	His	LBD	Beta 5
EphA5	Phe	132	Leu	LBD	Beta 7
EphA5	Phe	132	Cys	LBD	Beta 7
EphA5	Iso	736	Asn	Kinase	loop
EphA5	Iso	737	Asn	Kinase	Loop
EphA5	Leu	777	His	Kinase	C-terminal helix
EphA5	Leu	791	Pro	Kinase	C-terminal helix
EphA5	His	798	Asn	Kinase	Loop
EphA5	Ala	802	Thr	Kinase	C-terminal helix
EphA5	Pro	878	His	Kinase	Beta turn

EphA5	Pro	878	Arg	Kinase	Beta turn
EphA5	Iso	888	Asn	Kinase	C-terminal helix
EphA7	Iso	68	Lys	LBD	Beta 4
EphA7	Iso	149	Thr	LBD	Beta 9
EphA7	Leu	195	Ser	LBD	Beta 12
EphA7	Leu	628	Pro	Kinase	Loop
EphA7	Are	676	Ser	Kinase	N-terminal helix
EphA7	Val	707	Gly	Kinase	Beta 5
EphA7	Leu	749	Phe	Kinase	C-terminal helix
EphA7	Trp	931	Arg	SAM	Helix 1
EphA7	Trp	873	Cys	Kinase	C-terminal helix
EphA7	Iso	886	Arg	Kinase	C-terminal helix
EphB1	Gly	685	Cys	Kinase	Beta4
EphB1	Leu	721	His	Kinase	Alpha helix
EphB1	Val	741	Gly	Kinase	Beta C terminal
EphB1	His	742	Asn	Kinase	Beta turn
EphB1	Val	760	Ala	Kinase	Beta C terminal
EphB1	Gly	811	Ala	Kinase	Alpha helix
EphB2	Trp	72	Cys	LBD	Beta turn
EphB2	Trp	135	Cys	LBD	Beta turn
EphB2	Gly	638	Asp	Kinase	Beta 1
EphB2	Leu	711	His	Kinase	Helix
EphB2	Pro	795	Gln	Kinase	Helix
EphB2	Leu	860	Arg	Kinase	Helix
EphB3	Leu	710	Pro	Kinase	Beta5
EphB3	Leu	749	Gln	Kinase	C-terminal helix
EphB4	Trp	32	Cys	LBD	loop
EphB4	Phe	92	Cys	LBD	Beta 7
EphB4	Leu	166	Gln	LBD	Beta 10
EphB4	Phe	174	Cys	LBD	Beta 11
EphB4	Leu	731	Arg	Kinase	C-terminal Helix
EphB4	Val	748	Ala	Kinase	Beta turn C terminal
EphB4	Pro	820	Gln	Kinase	Beta turn C terminal
EphrinA2	Trp	112	Cys	RBD	Beta 6
EphrinB2	Iso	160	Asn	RBD	Loop
