# Supplementary material for: In-silico and structure-based assessment to evaluate pathogenicity of missense mutations associated with non-small cell lung cancer identified in the Eph-ephrin class of proteins
Source: Genomics Inform. 2023 Sep 27;21(3):e30. doi: 10.5808/gi.22069 (PMC10584653; doi:10.5808/gi.22069)
Supplement: Supplementary Table 2. — List of 32 mutations for different cancer types. [file gi-22069-Supplementary-Table-2.pdf]

Supplementary Table 2 – List of 32 mutations for different cancer types					
**S.No.**	**Protein**	**Mutation**	**Domain**	**Wild type PDB**	**Cancer type**
1.	EphA2	I619S	Kinase	4P2K	Ampullary carcinoma
2.	EphA2	F758C	Kinase	4P2K	Esophagogastric Adenocarcinoma
3.	EphA3	I109N	LBD	4L0P	Breast cancer
4.	EphA3	F152S	LBD	4L0P	Non-small cell lung cancer
5.	EphA3	I682N	Kinase	2QO2	Renal cell carcinoma
6.	EphA3	V688D	Kinase	2QO2	Esophagogastric cancer
7.	EphA3	A748T	Kinase	2QO2	Colorectal cancer
8.	EphA3	A749D	Kinase	2QO2	Non-small cell lung cancer
9.	EphA3	V762G	Kinase	2QO2	Esophagogastric cancer
10.	EphA3	W790C	Kinase	2QO2	Non-small cell lung cancer
11.	EphA4	L33S	LBD	2WO1	Renal Cell Carcinoma
12.	EphA4	L43H	LBD	2WO1	Esophageal squamous cell carcinoma
13.	EphA5	Y99H	LBD	4ET7	Endometrial carcinoma
14.	EphA5	F132L	LBD	4ET7	Esophagogastric Adenocarcinoma
15.	EphA5	F132C	LBD	4ET7	Esophagogastric Adenocarcinoma and Colorectal Adenocarcinoma
16.	EphA5	F132V	LBD	4ET7	Esophagogastric Adenocarcinoma
17.	EphA5	I736N	Kinase	2R2P	Endometrial carcinoma
18.	EphA5	I737N	Kinase	2R2P	Non-Melanoma
19.	EphA5	L791P	Kinase	2R2P	Head & Neck squamous cell carcinoma
20.	EphA5	H798N	Kinase	2R2P	Esophagogastric Adenocarcinoma
21.	EphA5	A802T	Kinase	2R2P	Pancreatic and Bladder Cancer
22.	EphA7	I68K	LBD	3NRU	Head & Neck squamous cell carcinoma
23.	EphA7	R676S	Kinase	2REI	Cervical Carcinoma
24.	EphA7	L749F	Kinase	2REI	Hepatobiliary Cancer and Non-Small Cell Lung Cancer
25.	EphB1	G685C	Kinase	3ZFX	Small cell lung cancer and non-small cell lung cancer
26.	EphB1	V741G	Kinase	3ZFX	Endometrial carcinoma
27.	EphB1	H742N	Kinase	3ZFX	Melanoma
28.	EphB1	V760A	Kinase	3ZFX	Mature B-Cell Neoplasms
29.	EphB3	L749Q	Kinase	3ZFY	Hepatocellular carcinoma
30.	EphB4	L731R	Kinase	6FNL	Melanoma
31.	EphB4	V748A	Kinase	6FNL	Lung squamous cell carcinoma

32.	EphrinA2	W112C	RBD	2WO3	Non-small cell lung cancer
