# Supplementary material for: In-silico and structure-based assessment to evaluate pathogenicity of missense mutations associated with non-small cell lung cancer identified in the Eph-ephrin class of proteins
Source: Genomics Inform. 2023 Sep 27;21(3):e30. doi: 10.5808/gi.22069 (PMC10584653; doi:10.5808/gi.22069)
Supplement: Supplementary Table 3. — Time-averaged secondary structure percentage analysis. [file gi-22069-Supplementary-Table-3.pdf]

Supplementary Table 3 – Time-averaged secondary structure percentage analysis

Secondary structure	Coil	B-Sheet	Bend	Turn	A-Helix
**Ephrin A2**	29	34	17	14	3
**W112C**	32	34	15	13	3
**EphA3 (KD)**	24	16	10	14	33
**A749D**	24	15	10	14	33
**W790C**	23	15	10	14	34
**EphA3 (LBD)**	23	46	16	1	2
**F152S**	23	46	16	1	2
**EphA7 (KD)**	22	16	13	14	29
**L749F**	23	15	11	12	34
**EphB1 (KD)**	23	15	12	13	33
**G685C**	24	15	12	14	31
**EphB4**	24	16	10	11	35
**V748A**	22	16	11	12	34
